# Caves of Vultures

**Published:** 1886-10

**Authors:** 


### *CAVES OF VULTURES.*

### Abodes of Death and Desolation Found in the Sespe Mountains.

A letter in the Ventura *Free Press* says: The writer has, for the last few years, offered a liberal reward for a set of vulture eggs, and numerous have been the attempts of both hunter and urchin to procure them, with one common result—they were unable to find where they nest. Believing that the Pacific condor nests somewhere in the Sespe Mountains, I determined to make one grand effort, if possible, to obtain the eggs. After considerable search I finally discovered the locality where these birds bring forth their young.

High up in one of the deep, dark gorges that put into the turbulent Sespe, a few miles above Devil's Gate, on the west side of the stream, at an altitude of more than four thousand feet, the home of the vulture was discovered, with all its strange and peculiar features. Climbing up the rocky gorge to a point where a perpendicular wall of rock fifty feet high stopped any further progress, and following along the base of the rocky cliff a few yards, I observed a cluster of pine trees that grew near the base of the cliff, and seeing one that shot above the projecting rock some sixty feet, being full of limbs that projected at right angles from the trunk, I determined to climb the tree and, if possible, get on top of the rocky shelf that, like a terrace, extended for hundreds of feet around the rocky bluff.

With but little exertion I climbed to a point parallel to the rocky shelf, and by careful attention walked out on one of the projecting limbs six or eight feet and stepped down upon the terrace. Here I found a rocky shelf some ten feet wide, and extending far along the bluff and back to an overhanging wall, arranged something after the manner of the Cliff-Dwellers of Arizona, in which were excavated chambers large enough for a person to comfortably pass in, being on an average ten feet deep.

On the floor of the caverns lie scattered around a very Golgotha of bones, while near the back I observed a pile of sticks, grass and other debris resembling a wood rat's nest, about three feet high, culminating in a uniformly pointed hillock. Taking a dry stick that lay near by, I proceeded to tear down this pile, believing that I had found at last a vulture's nest, and that the birds had covered up the eggs before departing in the morning. Denuding the pile a few inches, I came to the heads of half-decomposed carcasses of numerous small animals, in which I recognized the head of a pig, sheep, several jack rabbits and other small varmints. The stench was so great that I was compelled to retreat.

I entered a second chamber, where I found a similar pile, and pursuing my further investigation I entered a fourth one, containing one of those peculiar nests, in which I observed a hole at the base about six inches in diameter, and at the point of thrusting a stick into the hole, out came a most miserable looking creature, remaining for a moment and then darting back again. It once more appeared at the hole, completely filling it up, and this time I discovered that I was gazing upon a young vulture, and that these piles were in reality vultures' nests, the sole object of my perilous adventures. Stepping around into a second chamber I began to tear down one of those heaps, believing that I would find the eggs somewhere within the ghastly pile.

Just then, casting my eyes upward, I beheld far to the northward the old bird sailing high in the cloudless vault, and with renewed vigor I hastily demolished the pile, and when about one-third of the top had been torn off, consisting of partially decomposed carcasses of small animals, to my horror and surprise, there lay half buried in the heap, protruding from beneath, torn and lacerated, the hand and forearm of some human being, sufficiently intact not to be mistaken as to its identity. Appalled at this ghastly sight, and again looking to the northward, I beheld the old bird approaching the caverns.

Knowing as I did that they would swoop down in a few minutes, I sprang to the edge of a rock, seized a protruding limb and rapidly descended to the foot of the tree, just in time, for that moment, like a great avalanche, with the velocity of lightning, they came, shaking the very earth I stood upon. Simultaneously they alighted upon the projecting shelf, when, with their great wings still outstretched, they seemed to know at once that some daring intruder had ventured to enter their home of death and desolation.

Quickly wending my way down the mountain side, I finally reached my gun and outfit and casting a look in the direction of the cave, I beheld that there were unmistakable indications of turmoil in the camp—the great birds attacking each other for a moment in their imperial altitude, then alighting upon the overhanging rock, but too far distant for the range of my rifle. Hurriedly retracing my steps down the dismal and rocky stream, with the horrible and strange discoveries ever vividly before my eyes, it was not until I was past the Devil's Gate, where I halted, that I once more regained my normal condition of mind, determining that at some future day I would again visit the Vulture Caves of the Sespe.

